# Quantifying disparities in cancer incidence and mortality of Australian residents of New South Wales (NSW) by place of birth: an ecological study

**DOI:** 10.1186/s12889-015-2141-3

**Published:** 2015-08-26

**Authors:** Eleonora Feletto, Freddy Sitas

**Affiliations:** Cancer Research Division, Cancer Council NSW, 153 Dowling St, Woolloomooloo, NSW 2011 Australia; University of Sydney, School of Public Health, Sydney, Australia; University of NSW, School of Public Health and Community Medicine, Sydney, Australia

## Abstract

**Background:**

In 2013, about 32 % of the Australian population over 15 years of age was born overseas. Previous cancer-related immigrant health studies identified differences in mortality and incidence between immigrants and Australian-born people. To identify groups that may require targeted interventions, we describe by region of birth: 1. the highest cancer incidence and mortality rates for NSW residents, Australia’s most populous state; and 2. mortality to incidence ratios (MIR) for all cancers.

**Methods:**

Cancer incidence and mortality data were obtained from NSW residents for 2004–2008 (averaged) by sex, region of birth and 10 year age groups. Age standardised incidence and mortality rates were calculated with 95 % confidence intervals (per 100,000), using the world standard population. In the place of 5-year survival rates, we used age standardised MIRs (=M/I) as a simple proxy indicator of cancer survival.

**Results:**

All-cancer incidence only exceeded Australian born people (308.5) for New Zealand born (322). The highest reported incidence rates for cancers from all regions were prostate and breast cancers. All-cancer mortality exceeded Australian-born (105.3) in people born in Western Europe (110.9), Oceania (108.2) and UK and Ireland (106.4). For Australian-born residents, the MIR was 34 cancer deaths per 100 cases compared to residents from Central Europe at 38 deaths per 100 cases and lowest at 28 deaths per 100 cases for residents from Central and Southern Asia.

**Conclusion:**

Some disparities between Australian-born NSW residents and immigrants were identified in prostate, breast and lung cancer mortality rates. While on average most immigrant groups have similar cancer characteristics for the top cancers, areas for improvement to inform strategies to alleviate cancer disparities are required. This analysis suggests that NSW residents could benefit from specific prevention programmes on healthy eating and smoking cessation, especially people from Central Europe, UK and Ireland and Western Europe. Rising immigration rates encourage us to continue to address the areas indicated for improvement.

**Electronic supplementary material:**

The online version of this article (doi:10.1186/s12889-015-2141-3) contains supplementary material, which is available to authorized users.

## Background

In 2013, about 32 % of the Australian population over 15 years of age was born overseas, making its demographic composition culturally and linguistically diverse (CALD) [1]. Set to continue, projections to the year 2121 suggest that up to 280,000 people born overseas will settle in Australia per year [2]. The nation’s rising immigration and changing composition have multiple implications but our interest lies in the emerging health issues of immigrant communities. Previous research on immigrant health in Australia highlighted both positive and negative differences and encourage a greater understanding to reduce disparities [3–8] by developing an evidence based mechanism for health service planning as well as identifying CALD specific risk factors [6, 8]. The Australian and some state governments have launched initiatives specific to immigrant communities with a focus on modifying health service models [9–11], emphasising the reduction of the burden of chronic disease which complement the international healthy ageing agenda.

Cancer is one of the largest causes of chronic disease burden in Australia, resulting in approximately 30 % of all deaths in 2010 [12, 13]. With the exception of melanoma, Australians have comparable cancer patterns to other developed countries illustrated by the typically high rates of prostate, colorectal, lung and breast cancers. Previous cancer related immigrant health studies identified differences between immigrants and Australian-born people [6–8, 14, 15], and advocate for the monitoring of cancer incidence and mortality changes by population groups [6, 16]. Access to prevention services and treatment affects cancer burden and can accentuate disparities between immigrant groups especially given some cancer types are lifestyle related and could be prevented [17, 18]. On a national level, Anikeeva et al. [7] recently illustrated improved all-cancer mortality outcomes in Australian immigrants. However, the mortality rates for stomach, lung and bladder cancer increased from 1981–2007 [7].

Previous work by McCredie & Coates [19], McCredie et al. [20], Grulich et al. [8] and Supramaniam et al. [14] on cancer incidence in New South Wales (NSW), Australia’s most populous state, from the 1990s and early 2000s illustrated the issue of rising cancer incidence in immigrants from Asian regions, and the large burden of lung cancer attributed to people born in UK & Ireland. Nationally, incidence data have not been reported by place of birth and for NSW, analyses of cancer incidence and mortality rates have not been revised since the aforementioned studies. As a result, we chose to focus on cancer incidence and mortality rates by region of birth in NSW to identify any existing disparities between Australian-born and immigrant residents. Our primary aim is to describe cancer incidence and mortality for cancer sites with the highest rates by region of birth for NSW residents from 2004 to 2008. We also reported the mortality to incidence ratios (MIRs) for all cancers combined as a proxy for 5-year cancer survival rates.

## Methods

Cancer incidence and mortality data were obtained for all NSW residents by place of birth for 2004 to 2008 (averaged) by sex and 10 year age groups. Population figures were estimated by the NSW Ministry of Health based on Australian Bureau of Statistics (ABS) population figures. State based population data by immigrant group are available through official census reports which are not accessible on a yearly basis. Data on new cancer diagnoses and cancer deaths were obtained from the NSW Central Cancer Registry. The place of birth groupings used are in accordance with the Standard Australian Classification of Countries (SACC) adopted by the ABS [21]. Places of birth were grouped into 16 regions (Additional file 1: Table S1), broadly based on the Global Burden of Disease (GBD) project but collapsed as a result of small cell sizes and available population figures. The cell sizes did not allow for classification by SACC. The groupings represent the main CALD groups in NSW. As rates based on small counts tend to have poor statistical reliability, counts less than 16 and the corresponding rates were not reported, as is commonly done elsewhere [22, 23].

Incidence and mortality rates (reported as per 100,000 population) with 95 % confidence intervals by region of birth were age-standardised according to the direct method using the ‘World’ population [24]. Rates were calculated for the 20 cancer types with the highest incidence in NSW for 2008 [25] as well as liver and oesophageal cancers as they are known to be more prevalent in certain immigrant groups [14]. For brevity, only the five cancer types with the highest incidence and highest mortality rate by region of birth for all persons are reported in this analysis.

As immigration influxes are unpredictable, population pyramids for immigrant groups and thus mortality rates are fluid on a year-by-year basis, the calculation of survival rates by region of birth can be problematic. In the place of survival rates, we used a proxy indicator, age standardised MIRs (=M/I), as a simple predictor of 5-year survival rates [26]. These data were not available by time of immigration so stratification by length of time in NSW was not possible. All data collation, tabulation and calculations were performed by the Cancer Institute NSW, who provides these data on request. Neither ethics approval or informed consent were required.

## Results

Table 1 shows the overall incidence rates for the five cancer types with the highest incidence rates by region for all persons. All-cancer incidence rates exceeded those of Australian-born people (308.5) only for people born in New Zealand (322) and the highest incidence rates not exceeding that of Australian-born people, were in those from Western Europe (302.5), Oceania (301.6) and North America (293.4). The highest incidence rates for all regions were for prostate and breast cancers. Lung cancer was third highest for all but ‘Rest of African’ and South African-born NSW residents. Colon cancer was fourth highest for all but NSW residents born in Oceania, Rest of Africa and South Africa. Notably, melanoma of the skin is fifth highest for those born in Australia, New Zealand, UK & Ireland and fourth highest for South African-born people but did not appear in the top five incident cancers for any other regions. The only additional cancer type appearing in the top five for multiple regions was rectal cancer (North America, Central Europe, Western Europe, South Africa and Rest of Africa).

**Table 1:** Top 5 cancer types with the highest incidence and mortality rates by region (PERSONS): NSW residents averaged 2004-2008

	Australia			New Zealand			Oceania			Central and Southern Asia			East Asia			High Income Asia Pacific			Southeast Asia			UK & Ireland		
	ASR		95 % CI	ASR		95 % CI	ASR		95 % CI	ASR		95 % CI	ASR		95 % CI	ASR		95 % CI	ASR		95 % CI	ASR		95 % CI
INCIDENCE																								
ALL CANCERS	308.5		(306.6,310.5)	322		(308.3,336.1)	301.6		(267.2,337.8)	165.3		(153.1,178.1)	203.8		(194.3,213.5)	176.8		(169.9,192.6)	195.2		(186.6,204)	280.9		(273.6,288.3)
Prostate	1	119	(117.2,120.6)	1	108	(97.2,119.8)	2	70.9	(57.3,86.7)	2	52.8	(43.5,63.5)	2	42.2	(37,47.8)	2	48.8	(37.3,62.7)	2	49.8	(44.1,56.1)	1	94.2	(90.2,98.4)
Breast	2	81.9	(80.5,83.4)	2	91.4	(82.2,101.3)	1	92.2	(79.3,106.6)	1	59.3	(49.8,70)	1	57.2	(51.8,63)	1	49.8	(40.5,60.6)	1	62.7	(57.8,67.8)	2	83.6	(79,88.3)
Lung	3	26.8	(26.2,27.3)	3	30.8	(26.8,35.1)	3	28	(22.5,34.4)	3	12.1	(9.1,15.8)	3	24.8	(22.2,27.6)	3	14.7	(10.5,20.1)	3	18.3	(16.1,20.6)	3	30.4	(28.8,32.1)
Colon	4	25.8	(25.3,26.4)	4	29.1	(25.2,33.4)				4	11.3	(8.5,14.8)	4	20.4	(18,23)	4	14	(10,19.2)	4	14.4	(12.5,16.5)	4	20.8	(19.4,22.2)
Melanoma	5	25.3	(24.8,25.9)	5	18.7	(15.7,22.1)																5	14.1	(12.6,15.8)
Uterus							4	27.7	(20.6,36.4)															
Cervix							5	17.2	(11.9,24)															
Ovary										5	9.6	(6,14.5)												
Rectum													5	11.9	(10.1,14)									
Stomach																5	13.9	(9.9,18.9)						
Thyroid																			5	11.5	(9.9,13.2)			
MORTALITY																								
ALL CANCERS	105.3		(104.2,106.3)	104.7		(97.4,112.3)	108.2		(88.3,129.8)	46.8		(40.8,53.4)	66.2		(62,70.6)	62.1		(53.1,72.2)		67.1	(62.9,71.5)	106.4		(101.8,111.1)
Lung	1	20.8	(20.3, 21.3)	1	21.5	(18.2,25.2)	2	18.7	(14.2,24)	2	6.7	(4.5,9.5)	1	17.1	(15.1,19.4)	1	12.5	(8.6,17.5)	1	13.2	(11.4,15.2)	1	24.8	(23.4,26.3)
Breast	2	16	(15.4, 16.6)	2	17.6	(13.7,22.2)	1	19.1	(13.5,26.2)	1	14.9	(10.3,20.8)	4	5.7	(4.2,7.6)				2	7.7	(6,9.6)	2	15.4	(13.7,17.3)
Prostate	3	15.4	(14.8, 15.9)	3	15.1	(11.1,19.9)	3	17.6	(11, 26.6)										4	7	(5.1,9.4)	3	14	(12.7,15.4)
Colon	4	8.8	(8.5, 9.1)	4	8.6	(6.6,11)							3	5.7	(4.5,7.2)				5	5.1	(4,6.5)	5	7.1	(6.4,7.9)
CUP^a^	5	6.5	(6.2, 6.7)	5	7.7	(5.9,9.9)																4	7.4	(4.9,10.1)
Liver							4	6.4	(4, 9.6)				2	6.4	(5.1,7.9)	2	6.9	(4.1,10.9)	3	7.4	(6.1,8.9)			
Pancreas							5	5.9	(3.6, 9)				5	4.1	(3.1,5.4)									
Stomach																3	6.9	(4.1,10.7)						
	Western Europe			Eastern Europe			Central Europe			North East Africa & Middle East			Rest of Africa^b^		South Africa				North America			Latin America and Caribbean		
	ASR		95 % CI	ASR	95 % CI		ASR	95 % CI		ASR		95 % CI	ASR	95 % CI	ASR		95 % CI		ASR	95 % CI		ASR		95 % CI
INCIDENCE																								
ALL CANCERS	302.5		(292.1,313)	280.1		(249.3,312.5)	154.1		(145.5,162.9)	233.3		(223.6,243.3)	187.6		(165.9,211.1)	261.8		(242.4,282.3)	293.4		(270.4,317.6)	248		(230.3, 266.5)
Prostate	2	83.3	(79.5,87.3)	1	97.2	(75.3,122.1)	1	39.7	(35.8,44)	2	54.7	(49.4,60.5)	1	68.9	(52.5,88.6)	1	114.6	(96.0,135.7)	1	101.8	(83.7,122.4)	1	100.1	(85.8, 116.1)
Breast	1	84.4	(78.7,90.3)	2	78.6	(57.5,103.9)	2	35.2	(30.6,40.3)	1	72.2	(65.7,79.2)	2	64.1	(48.5,83)	2	94.2	(78.5,112)	2	100.9	(82.7,121.6)	2	74.2	(63.2, 86.5)
Lung	3	28.3	(26.6,29.9)	3	20.5	(14.3,27.7)	3	16.4	(14.6,18.4)	3	22.1	(19.7,24.8)	5	9.6	(5.6,15.4)				3	24.4	(17.7,32.6)	3	17.9	(13.8,22.7)
Colon	4	23.3	(21.8,24.9)	4	18.3	(12.7,24.9)	4	12.9	(11.2,14.7)	4	18.1	(15.8,20.5)	3	18.4	(12.6,25.9)	3	17.9	(13.1,24)	4	19.7	(14.1,26.7)	4	14	(10.6,18.3)
Melanoma																4	14.1	(9.9,19.5)						
Rectum	5	16.2	(14.9,17.7)				5	9.2	(7.7,10.8)				4	9.6	(5.6,15.5)	5	13.8	(9.7,18.9)						
Thyroid										5	12.7	(10.8,14.8)												
NHL				5	13.5	(7.6,21.2)													5	16.1	(11.1,22.5)			
Kidney																						5	12.1	(9.0,16.1)
MORTALITY																								
ALL CANCERS	110.9		(106.8,115.1)	95.7		(81.9,110.5)	59		(55.6,62.7)	82		(77.3,87)	62		(50.7,75)	79.9		(69.5,91.4)	96.9		(84.2,110.9)	77.8		(69.2,87.3)
Lung	1	21.7	(20.3,23.1)	1	19.6	(13.5,26.7)	1	11.8	(10.4,13.5)	1	17.5	(15.3,19.9)				3	8.9	(5.6,13.3)	1	19	(13.3, 26.4)	2	14	(10.5,18.3)
Breast	2	18	(15.5,20.6)	2	13.1	(5.5,24)	2	8.1	(6.1,10.4)	2	13.9	(11.1,17.1)				1	17	(10.7,25.5)	2	18.6	(11.0,29.3)	3	9.3	(5.7,14.3)
Prostate	3	11.3	(10.1,12.6)	4	7	(4,10.9)	3	6.2	(4.9,7.7)	3	7.7	(5.7,10.1)				2	15.1	(8.7,24.2)	3	14.9	(9.0,23.2)	1	17	(10.3,26)
CUP	5	7.3	(6.4,8.3)							5	5.7	(4.5,7.2)							5	6.9	(3.8,11.2)	5	5.4	(3.3,8.3)
Pancreas				3	7.6	(4.5, 11.4)				4	5.9	(4.7,7.3)							4	8	(4.7,12.6)			
Stomach				5	6.6	(3.1,11)	5	3.9	(3,5)													4	5.8	(3.6,8.7)

^a^CUP = cancer of unknown primary

^b^The region “Rest of Africa” does not include South Africa (a separate region) or Egypt and Tunisia (group with North East Africa and Middle East)

Table 1 also shows the overall mortality rates by place of birth and the five cancer types with the highest mortality rates by region. Overall mortality rates exceeded those of Australian-born (105.3) in people born in Western Europe (110.9), Oceania (108.2) and UK and Ireland (106.4). The highest mortality rates not exceeding that of Australian-born people were in those from New Zealand (104.7) and North America (96.9). The mortality rate for lung cancer was in the top five for all regions of birth (with the exception of Rest of Africa where the rate is not reported). Prostate, colon and breast cancers, and cancers of unknown primary were in the top five cancers by mortality across a number of regions.

**Fig. 1:**
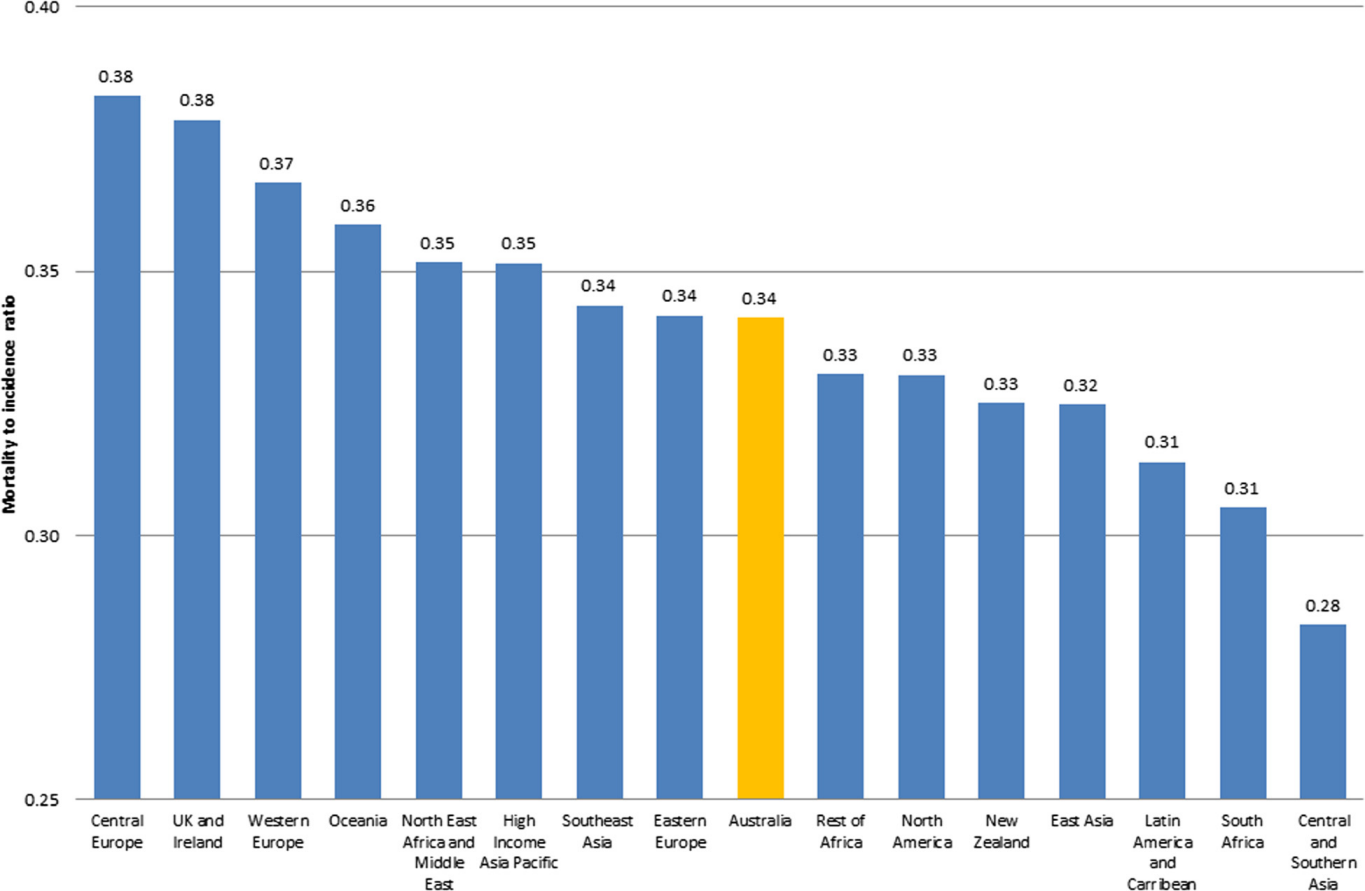
Mortality to Incidence Ratios for all cancers (persons)

The MIRs for all cancers were calculated to further assess the differences by region of birth (Fig. 1). For Australian-born NSW residents there were 34 deaths for every 100 new cases of cancer. This was highest for NSW residents from Central Europe at 38 deaths for every 100 new cases of cancer and lowest at 28 deaths for every 100 new cases of cancer for those from Central and Southern Asia.

## Discussion

Our findings indicate that most immigrant groups have a lower all-cancer incidence rate to Australian-born NSW residents, with the exception of New Zealand. The overall proportion of new cancer cases in immigrants rose from 24.5 % from 1991–2001 [6] to 29.2 % in 2004–2008, highlighting the growing health issue. The increase of new cases in immigrant groups is somewhat overshadowed by the consistently high incidence rates in Australian and New Zealand-born people. This is largely attributed to newly diagnosed breast and prostate cancer cases as well as melanoma, also apparent in international comparisons [15].

For breast and prostate cancers, current debate raises the issue of overdiagnosis [27], or diagnosis brought forward [28], which has contributed to inflated incidence rates due mammography use and PSA testing rates in Australia [15, 29, 30]. Analyses from the 45 and Up cohort study of over 250,000 NSW residents have shown that colorectal, breast and prostate cancer screening rates are generally lower for immigrant groups than for Australian-born people [31, 32]. However, as the years of living in Australia increased, the odds of screening approached the rates of Australian-born people [31]. As a result the incidence of prostate and breast cancers for immigrants may rise in the future, especially in groups recently immigrated to Australia, before mortality rates start declining.

The incidence rates of breast cancer across the different regions showed that the highest rates were in women born in western, typically English speaking, regions. An earlier study found similar breast cancer incidence rates in Australian-born women and women from high socio-economic countries in Asia but lower in women from low socio-economic Asian countries [8]. In contrast, our analysis found that women born in Southeast Asia had the highest breast cancer incidence amongst Asian-born women. The reason for this change from previous findings is not immediately clear and may point to some yet unmeasured risk, or that immigrants from these regions may not be always representative of the populations from which they have migrated.

The high rate of prostate cancer incidence globally in the Australian and New Zealand region [33] is also reflected in these NSW incidence rates. Prostate cancer incidence was lower in non-Australian born men than in Australian born-men, as previously shown [8, 14]. The driver of prostate cancer incidence may not be entirely due to PSA testing; Weber et al. [31] reported that PSA testing did not vary greatly for men in NSW by region of birth. Rather a recent analysis suggested that prostate cancer survival differences in NSW may be partially explained by geographic variations in patient management strategies, access to follow-up services and socio-economic disadvantage [34].

The incidence rates of colon cancer across the different regions showed that the highest rates were again in western, typically English-speaking, regions. Rectal cancer only appeared in the top five incident cancers for people born in ‘High Income Asia Pacific’. The federally funded National Bowel Cancer Screening Program (NBCSP), established in 2006, was only offered to people turning 50 and 55 years of age for the period of analysis [35]. The exact contribution of the NBCSP, offering faecal occult blood testing (FOBT), over this period is not clear. The ever screened rate for FOBT in the 45 + Up cohort was 23 % with a 39 % reduced risk of bowel cancer [36]. Although the data are not available by place of birth, it indicates that the NBCSP may have the capacity to reduce the incidence of colorectal cancer for all NSW residents. However there were lower reported screening rates in immigrant populations, which is not surprising considering its initial availability in English only [31, 32, 37]. Increased use of the NBCSP may temporarily increase the already higher incidence rates for English-speaking immigrants, but should also ultimately improve mortality outcomes. For Non-English speaking immigrants, revising the programme’s accessibility to include other CALD groups is important.

Our updated analysis confirms the previous evidence illustrating high lung cancer incidence across almost all immigrant population groups [8, 14, 19, 20]. Lung cancer incidence in NSW is higher in people born in UK & Ireland, New Zealand and Western Europe than those born in Australia. The smoking prevalence of immigrants from UK and Ireland and Western Europe for both men and women and New Zealand women is higher than Australian-born people [3]. Thus the continuing problem of high smoking prevalence and lung cancer incidence in these immigrant groups should be cause for concern and stimulate targeted preventive action to improve mortality outcomes associated with smoking related lung cancer.

The mortality rate for all cancers in NSW residents born in Oceania, UK and Ireland and Western Europe exceeds that of Australian-born people. This is a result of higher or comparable mortality rates in people from these regions of birth compared to Australian-born NSW residents for prostate, breast and/or lung cancers. Cancer risk factors may affect immigrants groups differently and studies of cancer mortality in immigrants suggest that there is broad convergence of lifestyles and associated incidence and mortality rate from that of the home country to the host country occurs after a number of years being resident in the host country [38]. Unfortunately analysis by number of years being resident was not possible as data relating to time of immigration was not available. This would suggest that cancer mortality rates in newly immigrated population groups will also rise in the future.

Nationally, breast cancer mortality rates are reportedly lower for the majority of immigrant women than for Australian-born women [7]. However, we found higher breast cancer mortality in women from New Zealand, Oceania, Western Europe, South Africa and North America than in Australian-born women. This may be reflective of the national decline in breast cancer mortality for Australian women where rates for women from other regions have stabilised [7]. Prostate cancer mortality rates in Australian men are notably higher than other developed countries, illustrating that high mortality rates for men regardless of their region of origin represents a broader issue potentially associated with the management of prostate cancer [29].

Colon cancer mortality was in the top five for 9 regions, highest in people born in Rest of Africa. On the other hand, the mortality rate for rectal cancer was not in the top five for any of the regions. Nationally, colorectal cancer mortality decreased for most immigrant groups from 1997–2007 with the exception of South Eastern European immigrants [7] and future reductions in mortality resulting from the implementation of the NBCSP are predicted [39].

Worth mentioning is the absence of cervical cancer as a top cause of mortality for women from any region. This is largely attributed to the success of the National Cervical Screening Program established in 1991. However, it was one of the top five incident cancers for women from Oceania [17] who may, therefore, benefit from targeted encouragement to participate in screening. These findings are not yet influenced by the recent roll out of the human papillomavirus (HPV) vaccine through the National HPV Vaccination Program and, therefore, it is assumed that incidence and mortality rates for cancer of the cervix will continue to improve in the future.

Lung cancer has one of the highest mortality rates for all but one region. However, the age-standardised rate for lung cancer was only higher for people from UK & Ireland compared to Australian-born people. Lung cancer mortality rates of people from Asian regions have decreased since the early 1980s [7] and, in this study, NSW residents from Asian regions also had lower lung cancer mortality rates than Australian-born. The high smoking prevalence of people from Asian countries is not reflected in NSW [3, 40] and could have contributed to the lower lung cancer incidence and mortality rates seen here. As a major cause of lung cancer is smoking, targeting cessation programmes to smokers born in the UK and Ireland, New Zealand and Western Europe could reduce the overall lung cancer burden.

The MIRs indicated that NSW residents born in Central Europe were worse off. The case mix of cancers in the top five for Central Europe born people was similar to Australian born people with the additional inclusion of rectal cancer. For mortality, stomach cancer was one of the top five cancers for people born in Central Europe. This is consistent with previous findings which highlighted stomach cancer as having worse outcomes in immigrant groups compared to Australian born people [7].

Incidence and mortality rates for people born in Asian regions are somewhat contradictory with previous findings, indicating rising cancer incidence. In the absence of updated trend data, the MIRs illustrate that people from ‘High Income Asia Pacific’ and Southeast Asia still exhibit worse cancer outcomes than Australian-born people. The case mix indicates that this could be driven by stomach cancer incidence and mortality. As well as its infectious aetiology [41], dietary factors namely consumption of salt and salty foods also have a probable association to increased stomach cancer risk [42], which has been noted in the dietary habits of people from these regions. Thyroid cancer incidence for Southeast Asia-born people and liver cancer mortality for High Income Asia Pacific-born people also contribute to the cancer burden for immigrants from these regions. These data allow for identification of specific population groups requiring further exploration and attention. That is, a targeted analysis of people from Central Europe, UK and Ireland and Western Europe, the regions with the highest MIR, could inform specific programmes on healthy eating, smoking cessation and other prevention initiatives to improve outcomes. The reverse is also applicable, in that an analysis of regions with low MIRs may highlight beneficial differences.

The mortality to incidence results are difficult to interpret unless formal analyses of survival are conducted, bearing in mind case mix per population, and the limitation of shifting annual population denominators. If MIRs are a crude reflection of survival, then they may indicate health service needs that require further attention. However, we acknowledge that the population-region specific MIRs may overestimate any survival benefit and are included here as indicators of regional immigrant groups that require further attention. For example, cancers with poor prognosis were shown to have high mortalities in immigrants from many parts of Asia but are masked by cancers with better prognoses when assessing the corresponding MIRs which were low.

In NSW, complete registration of cancer cases and deaths with the NSW Central Cancer Registry are mandated under the Public Health Act 2010. As a result, high quality data are available and place of birth is also routinely collected. However, the population figures by place of birth are subject to significant fluctuations as a result of changing immigration trends. To allow for this, we chose to average figures for a five year period and group places of birth into bigger groupings. However, the GBD groupings combine countries which may give rise to generalisations which could mask important lifestyle differences. Additionally, some groupings artificially divide geographic locations but efforts were made to mirror the classifications of the Global Burden of Disease Project within the limitations of available population by place of birth information. Only data up to 2008 were used, despite 2009 incidence figures being available, to ensure comparability with mortality data. Trend analysis was not conducted due to data availability and its relevance considering the previous work conducted [7, 14]. However, there are only limited data available on immigrant groups in NSW and this analysis highlight, variations in cancer incidence and mortality that could be lifestyle related and a rich and diverse population for future aetiological studies [6]. We have avoided direct comparisons of cancer rates with countries of origin because, as we described previously [6], immigrants are on average healthier and often economically more advantaged than their homeland population. Further, rates from cancer registries are not always representative of country averages, because not all countries have national coverage. Rather, we provided World-standardised rates for future careful comparisons bearing these caveats in mind.

## Conclusions

While this analysis showed that immigrant groups do not fare badly, rising immigration rates encourage us to continue to address the areas indicated for improvement. Disparities in cancer incidence or mortality between population groups and Australian-born were identified in this analysis especially in prostate, breast and lung cancer mortality rates. It is important to disseminate this type of information so we are able to drive targeted research into effective strategies to alleviate health disparities. Analysing cancer outcomes by region of birth aids in the planning of health care services for these communities and assist in the future study of aetiological factors associated with individual cancer types. In a context where government is reining in health care spending, addressing health disparities, especially in immigrant groups, will result in long term budgetary savings and better health outcomes [43]. While previous studies have rightly focused on non-English CALD groups, it seems the lifestyle of people born in mainly English speaking countries has been overlooked, and does appear to contribute to a large lung cancer burden, much of it likely to be attributed to smoking. The observed variation in cancer patterns among population groups indicates the importance of monitoring these groups separately, as these patterns may provide aetiologic clues that could be investigated by further studies.

## Additional file

Additional file 1: Table S1. Region of birth groupings (DOCX 16 kb)

## References

[1] Australian Bureau of Statistics (2014). 6250.0 - Characteristics of recent migrants, Australia, Nov 2013.

[2] Australian Bureau of Statistics (2013). 3222.0 - Population projections Australia 2012 (BASE) to 2101.

[3] Weber MF, Banks E, Sitas F (2011). Smoking in migrants in New South Wales, Australia: report on data from over 100 000 participants in The 45 and Up Study. Drug Alcohol Rev.

[4] Anikeeva O, Bi P, Hiller JE, Ryan P, Roder D, Han G-S (2015). Trends in migrant mortality rates in Australia 1981–2007: a focus on the national health priority areas other than cancer. Ethn Health.

[5] Al Abed NA, Davidson PM, Hickman LD (2014). Healthcare needs of older Arab migrants: a systematic review. J Clin Nurs.

[6] Supramaniam R, O’Connell D, Robotin M, Tracey E, Sitas F (2008). Future cancer trends to be influenced by past and future migration. Aust N Z J Public Health.

[7] Anikeeva O, Bi P, Hiller JE, Ryan P, Roder D, Han G-S (2012). Trends in cancer mortality rates among migrants in Australia: 1981–2007. Cancer Epidemiol.

[8] Grulich AE, McCredie M, Coates M (1995). Cancer incidence in Asian migrants to New South Wales. Australia Br J Cancer.

[9] Australian Government Department of Health and Ageing (2012). National ageing and aged care strategy for people from Culturally and Linguistically Diverse (CALD) backgrounds.

[10] NSW Ministry of Health (2012). NSW Health policy & implementation plan for culturally diverse communities 2012–2016.

[11] Victorian Government (2011). Victorian health priorities framework 2012–2022: metropolitan health plan.

[12] Begg S, Vos T, Barker B, Stevenson C, Stanley L, Australian Institute of Health and Welfare (2007). The burden of disease and injury in Australia 2003.

[13] Australian Institute of Health and Welfare (2012). Australia’s health 2012.

[14] Supramaniam R, O’Connell D, Tracey E, Sitas F (2006). Cancer incidence in new south wales migrants 1991–2001.

[15] Australian Institute of Health and Welfare (2012). Cancer in Australia: an overview.

[16] McKay L, Macintyre S, Ellaway A (2003). Migration and health: a review of the international literature.

[17] Gushulak BD, MacPherson DW (2006). The basic principles of migration health: Population mobility and gaps in disease prevalence. Emerg Themes Epidemiol.

[18] Schottenfeld D, Beebe-Dimmer JL, Buffler PA, Omenn GS (2013). Current perspective on the global and United States cancer burden attributable to lifestyle and environmental risk factors. Annu Rev Public Health.

[19] McCredie M, Coates M (1989). Cancer incidence in migrants to New South Wales: 1972 to 1984.

[20] McCredie M, Coates M, Duque Portugal F, Smith DP, Taylor R (1993). Common cancers in migrants to New South Wales 1972–1990.

[21] Australian Bureau of Statistics (2008). 1269.0 Standard Australian Classification of Countries (SACC) Australia.

[22] Brillinger DR (1986). The natural variability of vital rates and associated statistics. Biometrics.

[23] Fay MP, Feuer EJ (1997). Confidence intervals for directly standardized rates: a method based on the gamma distribution. Stat Med.

[24] Segi M (1960). Cancer mortality for selected sites in 24 countries (1950–57).

[25] Tracey E, Kerr T, Dobrovic A, Currow DC (2010). Cancer in NSW: incidence and mortality report 2008.

[26] Asadzadeh Vostakolaei F, Karim-Kos HE, Janssen-Heijnen MLG, Visser O, Verbeek ALM, Kiemeney LALM (2011). The validity of the mortality to incidence ratio as a proxy for site-specific cancer survival. Eur J Public Health.

[27] Vickers AJ, Sjoberg DD, Ulmert D, Vertosick E, Roobol MJ, Thompson I (2014). Empirical estimates of prostate cancer overdiagnosis by age and prostate-specific antigen. BMC Med.

[28] Independent UK Panel on Breast Cancer Screening (2012). The benefits and harms of breast cancer screening: an independent review. Lancet.

[29] Feletto E, Bang A, Cole-Clark D, Chalasani V, Rasiah K, Smith D. An examination of prostate cancer trends in Australia, England, Canada and USA: Is the Australian death rate too high? World J Urol. 2015;Feb 20 [Epub]. 10.1007/s00345-015-1514-7 PMC4617845 25698456

[30] Barratt A (2015). Overdiagnosis in mammography screening: a 45 year journey from shadowy idea to acknowledged reality. BMJ.

[31] Weber MF, Banks E, Smith DP, O’Connell D, Sitas F (2009). Cancer screening among migrants in an Australian cohort; cross-sectional analyses from the 45 and Up Study. BMC Public Health.

[32] Weber MF, Chiew M, Feletto E, Kahn C, Sitas F, Webster L (2014). Cancer screening among immigrants living in urban and regional australia: results from the 45 and up study. Int J Environ Res Public Health.

[33] Ferlay J, Soerjomataram I, Ervik M, Dikshit R, Eser S, Mathers C, et al. GLOBOCAN 2012 v1.0 [Internet]. Cancer Incidence and Mortality Worldwide. IARC CancerBase No. 11 [Internet]. Lyon, France: International Agency for Research on Cancer. 2013 [cited 2013 Dec 17]. Available from: http://globocan.iarc.fr/Default.aspx

[34] Yu XQ, Luo Q, Smith DP, O’Connell DL, Baade PD (2014). Geographic variation in prostate cancer survival in New South Wales. Med J Aust.

[35] Australian Government Department of Health. Screening - About the Program [Internet]. National Bowel Cancer Screening Program. 2014 [cited 2014 Jul 1]. Available from: http://www.cancerscreening.gov.au/internet/screening/publishing.nsf/Content/about-the-program-1

[36] Steffen A, Weber MF, Roder DM, Banks E (2014). Colorectal cancer screening and subsequent incidence of colorectal cancer: results from the 45 and Up Study. Med J Aust.

[37] Christou A, Katzenellenbogen JM, Thompson SC (2010). Australia’s national bowel cancer screening program: does it work for indigenous Australians?. BMC Public Health.

[38] Kliewer EV, Smith KR (1995). Breast cancer mortality among immigrants in Australia and Canada. J Natl Cancer Inst.

[39] Grogan PB, Olver IN (2014). A bowel cancer screening plan at last. Med J Aust.

[40] Eriksen M, Mackay J, Ross H (2012). The tobacco atlas.

[41] IARC Working Group on the Evaluation of Carcinogenic Risks to Humans (2009). IARC Monographs: biological agents.

[42] Fund WCR (2007). American institute for cancer research. Food, nutrition, physical activity, and the prevention of cancer: a global perspective.

[43] Zimmerman C, Kiss L, Hossain M (2011). Migration and health: a framework for 21st century policy-making. PLoS Med.

