# Supplementary material for: Quantifying disparities in cancer incidence and mortality of Australian residents of New South Wales (NSW) by place of birth: an ecological study
Source: BMC Public Health. 2015 Aug 26;15:823. doi: 10.1186/s12889-015-2141-3 (PMC4548689; doi:10.1186/s12889-015-2141-3)
Supplement: Additional file 1: Table S1. — Region of birth groupings (DOCX 16 kb) [file 12889_2015_2141_MOESM1_ESM.docx]

Supplementary Table 1: Region of birth groupings

| Region of birth | Countries or places included in region |
| --- | --- |
| Australia | Australia, Australian external territories, Norfolk Island, Antarctica |
| New Zealand | New Zealand |
| Oceania | Melanesia, New Caledonia, Papua New Guinea, Solomon Islands, Vanuatu, Kiribati, Palau, Cook Islands, Fiji, Niue, Samoa, American Samoa, Tokelau, Tonga, Tuvalu, Polynesia (excludes Hawaii), Adélie Land, Argentinian Antarctic Territory, British Antarctic Territory, Chilean Antarctic Territory |
| High Income Asia Pacific | Singapore, Japan, Republic of Korea |
| Southeast Asia | Burma (Myanmar), Cambodia, Laos, Thailand, Vietnam, Brunei Darussalam, Indonesia, Malaysia, Philippines, East Timor, Maldives, Sri Lanka |
| Central and Southern Asia | Bangladesh, India, Nepal, Pakistan, Afghanistan, Armenia, Azerbaijan, Georgia, Kazakhstan, Uzbekistan |
| East Asia | China, Hong Kong, Macau, Taiwan, Democratic People's Republic of Korea |
| Central Europe | Albania, Bosnia and Herzegovina, Bulgaria, Croatia, Former Yugoslav Republic of Macedonia, Romania, Slovenia, Montenegro, Serbia, Czech Republic, Hungary, Poland, Slovakia |
| Eastern Europe | Moldova, Belarus, Estonia, Latvia, Lithuania, Russian Federation, Ukraine |
| Western Europe | Austria, Belgium, France, Germany, Netherlands, Switzerland, Denmark, Finland, Iceland, Norway, Sweden, Holy See, Italy, Malta, Portugal, Spain, Cyprus, Greece, Israel |
| UK and Ireland | Channel Islands, England, Isle of Man, Northern Ireland, Scotland, Wales, Ireland |
| North East Africa and Middle East | Egypt, Tunisia, Bahrain, Gaza Strip and West Bank, Iran, Iraq, Jordan, Kuwait, Lebanon, Saudi Arabia, Syria, Turkey, United Arab Emirates, Yemen |
| West, Southern and Eastern Africa (referred to in text as Rest of Africa) | Algeria, Morocco, Sudan, Chad, Congo, Democratic Republic of Ghana, Libya, Liberia, Niger, Nigeria, Sierra Leone, Togo, Southern and East Africa, Angola, Ethiopia, Kenya, Malawi, Mauritius, Mozambique, Namibia, Réunion, Tanzania, Uganda, Zambia, Zimbabwe |
| South Africa | South Africa |
| North America | Americas, Canada, United States of America |
| Latin America and Caribbean | Bermuda, Argentina, Bolivia, Brazil, Chile, Colombia, Ecuador, Falkland Islands, Guyana, Paraguay, Peru, Uruguay, Venezuela, Costa Rica, El Salvador, Guatemala, Mexico, Nicaragua, Caribbean, Bahamas, Barbados, Cayman Islands, Cuba, Grenada, Jamaica, Netherlands Antilles, St Vincent and the Grenadines, Trinidad and Tobago |
